# Gastric alarmin release: A warning signal in the development of gastric mucosal diseases

**DOI:** 10.3389/fimmu.2022.1008047

**Published:** 2022-10-06

**Authors:** Enqin Wu, Jiaxing Zhu, Zhiyuan Ma, Biguang Tuo, Shuji Terai, Kenichi Mizuno, Taolang Li, Xuemei Liu

**Affiliations:** ^1^ Department of Gastroenterology, Digestive Disease Hospital, Affiliated Hospital of Zunyi Medical University, Zunyi, China; ^2^ Division of Gastroenterology & Hepatology, Graduate School of Medical and Dental Sciences, Niigata University, Niigata, Japan; ^3^ Department of General Surgery, Affiliated Hospital of Zunyi Medical University, Zunyi, China

**Keywords:** gastric alarmin, IL-33, high mobility group (HMG) family, antimicrobial peptides (AMPs), defensin, cathelicidin, gastric mucosal diseases

## Abstract

Alarmins exist outside cells and are early warning signals to the immune system; as such, alarmin receptors are widely distributed on various immune cells. Alarmins, proinflammatory molecular patterns associated with tissue damage, are usually released into the extracellular space, where they induce immune responses and participate in the damage and repair processes of mucosal diseases.In the stomach, gastric alarmin release has been shown to be involved in gastric mucosal inflammation, antibacterial defense, adaptive immunity, and wound healing; moreover, this release causes damage and results in the development of gastric mucosal diseases, including various types of gastritis, ulcers, and gastric cancer. Therefore, it is necessary to understand the role of alarmins in gastric mucosal diseases. This review focuses on the contribution of alarmins, including IL33, HMGB1, defensins and cathelicidins, to the gastric mucosal barrier and their role in gastric mucosal diseases. Here, we offer a new perspective on the prevention and treatment of gastric mucosal diseases.

## Introduction

Alarmins are endogenous, constitutive molecules that are rapidly released from cells in response to infection or tissue damage; thus, alarmins act as early warning signals to the immune system by promoting the chemoattraction of antigen-presenting cells and activating innate and adaptive immunity (1). Alarmins are distinct from microbial pathogen-associated molecular patterns (PAMPs); rather, alarmins are derived from host cell damage-associated molecular patterns (DAMPs) generated upon host cell injury and bind to pattern recognition receptors (PRRs) of the innate immune system, thereby initiating downstream inflammatory responses (2, 3). Under normal circumstances, alarmins are present in intracellular granules, the nucleus or the cytoplasm and are involved in protein regulation (4, 5). After alarmins are rapidly released into the extracellular space in response to stimuli such as degranulation, cell death, and induction, receptor-expressing immune cells are recruited and activated, which can help restore immune homeostasis and promote epithelial repair (6); however, alarmin release can also lead to persistent tissue damage (7, 8). All alarmins have the ability to promote inflammation and immunity, but these factors play different roles after recognition by different receptors (1, 9–12). Alarmins are involved in processes such as inflammation, antibacterial defense, adaptive immunity, and wound healing and result in tissue damage and repair, highlighting the potential value of alarmins (13–19).

Alarmins are diverse and can be generally classified as antimicrobial peptides (AMPs), nuclear binding proteins, heat shock proteins (HSPs), ionic binders, nucleotides/metabolites (adenosine triphosphate and uric acid) and extracellular matrix degradation products (20–24). However, some alarmin studies revealed that nuclear binding proteins, including IL-33 (25–27) and high mobility group B1 (HMGB1) (28), and AMPs, including defensins (29, 30) and cathelicidin (31, 32), were mainly involved in the development of gastric mucosal diseases.

**Figure 1:**
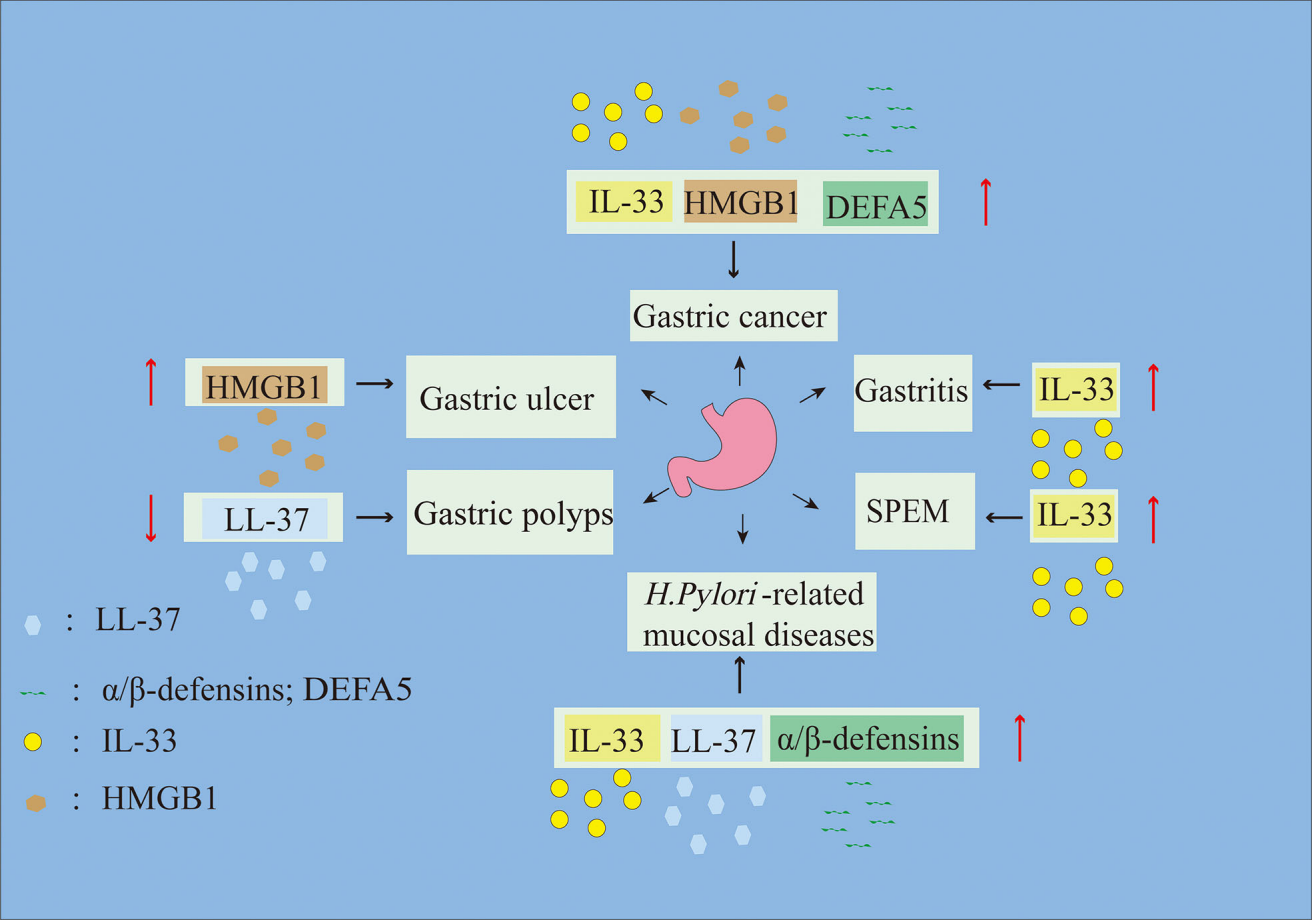
Alarmins are involved in the progression of gastric mucosal diseases. Up arrows represent alarmin upregulation, and down arrows represent alarmin downregulation.

Gastric mucosal diseases involve mucosal tissue damage and include various types of gastritis, gastric ulcers (GUs) and gastric tumors (33–35). Recent studies have shown that alarmins are highly expressed in gastric mucosal diseases and initiate the close cooperation between innate and adaptive immunity to regulate host damage and repair (36). Therefore, this review mainly focuses on the pathophysiological roles of alarmins in gastric mucosal injury and disease (**Figure 1**; **Table 1**) to provide new insights into gastric mucosal diseases.

**Table 1:** Multiple gastric alarmins involved in the development of gastric mucosal diseases are mentioned in this review.

Gastric alarmins	Type	Localization	Receptor	Immune cells activated/recruited by alarmins	Physiological function	Relationship with gastric mucosal diseases
Nuclear binding proteins	IL-33	Gastric surface mucous cell nucleus (29).	ST2 receptor (29).	Th1 cells, Th2 cells, cytotoxic T cells, Treg cells, ILC2s, eosinophils, basophils, NK cells, mast cells (37–55)	Regulate gene expression and maintain gastric barrier function (5) (35) (56, 57).	IL-33 upregulation promotes the development of chronic atrophic gastritis, SPEM, GC (58) (59–64) (65, 66). IL-33 upregulation promotes SPEM formation after acute gastric injury (64) (67–69). IL-33 upregulation is associated with injury and repair in *H. pylori*-associated gastritis (6, 27, 70, 71) (72).
	HMGB1	The nucleus and cytoplasm of gastric epithelial cells (30).	TLR, RAGE (73–77).	Dendritic cells, T cell (78)	Regulate gene transcription and maintain immune homeostasis in the gastric mucosa (79–81).	HMGB1 promotes gastric ulcer and GC progression and exacerbates mucosal damage (82–84) (85) (86–88).
Antimicrobial peptides	Defensins	Gastric epithelial cell membrane (89–91).	CCR2, CCR6, TLR4, TLR9 (92–94).	Immature DCs, monocytes and T cell (95–97).	Antibacterial and Maintain gastric mucosal immune homeostasis (90) (98, 99).	DEFA5 upregulation inhibits the development of GC (100). Downregulation of defensins promotes the progression of Helicobacter pylori-associated gastritis (30, 101–103).
	Cathelicidin	The cytoplasm of surface epithelial cells, chief cells and parietal cells of the fundic gland (104).	FPRL1, TLR7-9 (105–108).	Neutrophils, Monocytes, Plasmacytoid, myeloid DCs (109, 110).	Inhibit *H. pylori* and regulate gastric mucosal immune function (111, 112).	Catherin deficiency promotes the development of *H. pylori-*associated gastritis, gastric hyperplastic polyps and GC (104, 113) (111, 114),.

## Gastric alarmin nuclear binding proteins: IL-33 and HMGB1

Nuclear binding proteins are a class of DNA-binding proteins that are involved in the regulation of transcription, replication and repair (37). Alarmins classified nuclear binding proteins in the stomach include IL-33 (38) and HMGB1 (39, 40), which are released extracellularly, causing immune changes and participating in the development of gastric mucosal diseases.

### The expression pattern and functional role of IL-33 in the stomach

IL-33 is a nuclear binding protein in the IL-1 cytokine family (6, 41). Under physiological conditions, IL-33 translocates to the nucleus and is involved in regulating gene expression (5) and the maintenance of barrier function (42). The alarmin IL-33 is released in response to epithelial barrier damage or cell necrosis (43, 65) and signals through the IL-33 receptor complex, which consists of interleukin-1 receptor-like 1 (ST2) and interleukin-1 receptor accessory protein (IL1RAcP) (38). These receptors are widely expressed on various immune cells, including T helper type 2 (Th2) cells (44–46), activated T helper type 1 (Th1) cells (47), cytotoxic T cells (48, 49), type 2 innate lymphocytes (ILC2s) (50–54), eosinophils (55, 115), basophils (56, 116), natural killer (NK) cells, NKT cells (57), mast cells (MCs) (58, 117–119) and Treg cells (120). Therefore, these factors are involved in coordinating immune defense and repair mechanisms (121) and trigger adaptive immune responses (122).

In the stomach, IL-33 is highly expressed and localized to the nuclei of a subset of foveolar cells (also known as surface mucous cells (SMCs) or pit cells) (38), but a small fraction of IL-33 positive cells colocalize with Ki-67. This finding suggests that IL-33 expression changes as progenitors differentiate into pre-SMCs; specifically, surface IL-33 expression is inhibited as SMCs mature and migrate (65). Recent studies have shown that IL-33, which is released extracellularly, is involved in gastric injury and repair (36). In addition, recent studies have identified IL-33 as a DAMP that signals innate immune cells in response to stress or cell membrane disruption (123, 124) and that participates in the development of gastric mucosal diseases (65).

### IL-33 release is involved in the development of gastric mucosal diseases

#### IL-33 upregulation promotes the development of chronic atrophic gastritis, gastric metaplasia and gastric cancer

Chronic atrophic gastritis, spasmolytic polypeptide-expressing metaplasia (SPEM) and GC are states along the continuous developmental trajectory of gastric mucosal disease, and IL-33 is a potential promoter of this process. IL33 is mainly induced by chronic phosphorylation of signal transducer and activator of transcription 3 (STAT3), and previous studies have shown that overactivated STAT3 is involved in gastric inflammation (59) and can lead to Th2-mediated gastric metaplasia (65). Gastric metaplasia includes SPEM (60, 61) and intestinal metaplasia. In SPEM, chief cells transdifferentiate into cells with a mucinous metaplastic phenotype, usually in the presence of parietal cell atrophy, epithelial barrier impairment, and ulceration (62–64, 125–127), and express Muc6 and TFF2 at the base of the gland (128). These findings suggest that SPEM develops as a physiological healing response after injury through the transdifferentiation of zymogen-secreting cells into mucinous cell metaplasia. Furthermore, studies in a long-term adrenalectomy SPEM model (129) have shown that SPEM progression is delayed in only IL-33 KO mice and that treatment of mice with recombinant IL-33 induces gastritis with a potential SPEM phenotype (65). Thus, IL-33 may be an important initiator of SPEM development. However, another study reported (66) that IL-13 KO mice are resistant to SPEM but still exhibit immune cell infiltration, possibly due to limitations of the L635-induced acute SPEM model. However, IL-33 is critical for promoting signaling through the downstream factor IL-13 to drive the induction of metaplasia (66); notably, the IL-33 and IL-13 cytokine signaling networks promote SPEM by driving M2 macrophage polarization (130) and affect mitochondrial metabolism (131). These findings were validated in mouse models and in human metaplastic tissues; one study confirmed that M2 macrophages promote SPEM expansion and intestinal metaplasia in the presence of inflammation and parietal cell shrinkage (130), and other studies validated that M2 macrophages promote SPEM progression (66, 130). In addition, IL-33 can affect many immune cell types to regulate SPEM development. ILC2s play an important role in this process, as gastric immune cell infiltration and SPEM are largely blocked by ILC2 depletion (65, 67), suggesting that these cells may be the main responders to gastric IL-33 release. However, some researchers have suggested that MCs may drive SPEM development through IL-13 (68), and IL-33 is upstream of IL-13 (66). Therefore, the IL-13-mediated activation of ILC2s or MCs may be a complementary mechanism by which immune cells induce SPEM development. In addition, it has been suggested that eosinophils contribute to SPEM (25), although some researchers believe these cells are dispensable (69). However, the depletion of eosinophils significantly reduces local IL-33-producing M2 macrophages and SPEM (25). Thus, IL-33 is a key mediator of chronic gastritis (CG) and intestinal metaplasia that promotes metaplasia and M2 macrophage-dependent eosinophilic infiltration, leading to SPEM progression (25). These data suggest that IL-33 is a potential therapeutic target for precancerous lesions of the gastrointestinal tract.

Furthermore, the metaplastic program can be perpetuated by persistent injury and chronic inflammatory stimulation, leading to the transformation to GC (36). Some researchers have shown that IL-33 can promote continuous GC cell growth by inducing the MC-dependent production and release of macrophage-attracting factors (27) or by activating the mitogen-activated protein kinase (MAPK) pathway, which includes extracellular signal-regulated kinases (ERKs) such as ERK1/2, JNK and p38 (70, 71), thereby promoting GC proliferation, differentiation, migration and apoptosis (132). Interestingly, a recent study showed that IL-33 can inhibit cyclin C (CCNC, G0/G1 transition) and cyclin B1 (CCNB1, G2/M transition) and that cysteine aspartase-3 (CASP3) activation decreases tumor cell proliferation and thus may be involved in cell proliferation in an environment- and cell type-dependent manner (72). Therefore, IL-33 may be an immunotherapy target for preventing the progression of CG to early stage GC.

Moreover, it is worth mentioning that IL-33 promotes the development of SPEM upon acute gastric mucosal injury (67). The occurrence of SPEM at the ulcer margins of regenerating gastric glands (63, 133) represents a repair process after acute mucosal injury (63, 134). Moreover, recent studies have shown that IL-33 mRNA is rapidly upregulated in acute gastric injury through TFF2 and phosphorylated ERK1/2, while the activation of ERK1/2 and the expression of the innate immunity cytokines IL1α, IL1β, and IL6 are increased (65), suggesting that IL-33 can mediate acute inflammatory injury in the stomach. IL-33 is released into the extracellular environment, and the IL-33 receptor engages a signaling pathway as a key regulator of SPEM development, thereby promoting cytokine and immune regulation in response to acute gastric epithelial injury (66). Mice lacking IL-33 or subunits of the IL-33 receptor complex fail to develop SPEM following acute parietal cell loss (66). Therefore, the gastric alarmin IL-33 plays an important role in gastric focal and diffuse injury, and alterations in IL-33 result in the development of gastric mucosal diseases.

#### IL-33 is involved in the occurrence of *Helicobacter pylori* (*H. pylori*)-related mucosal diseases

Recent studies have suggested that IL-33 may be an alarmin in *H. pylori*-positive patients (135). IL-33 is highly expressed in the mucosa of patients with *H. pylori*-infected gastritis (136), and ST2 is recruited into membrane rafts in response to IL-33 release by *H. pylori*-infected gastric epithelial cells (137), which promotes the production of tumor necrosis factor-α (TNF-α) by MCs and inhibits the proliferation of gastric epithelial cells, leading to the progression of *H. pylori*-associated gastritis and bacterial colonization (138). Moreover, one study found that IL-33 can regulate the phenotype and activity of MCs. IL-33 stimulates the expression of the Dectin-1, Dectin-2, RIG-I and nucleotide-binding oligomerization domain-containing protein 1 (NOD1) receptors in mature MCs to enhance and modulate the inflammatory response, perhaps stimulating MCs to release numerous proinflammatory and immunomodulatory factors and induce migratory responses during *H. pylori* infection (139). However, another study showed that IL-33 expression increases during acute *H. pylori* infection and may promote gastric mucosal regenerative activity through collagen I (140). This finding suggests that IL-33 not only represents damage in *H. pylori*-related gastric mucosal disease but also may be involved in tissue repair processes. IL-33 is released into the extracellular environment, where it has been shown to recruit immune cells to enhance mucosal immune defense and repair mechanisms. IL-33 attracts circulating innate immune cells by activating resident IL-1R4+ (ST2) MCs and dendritic cells (DCs) and releasing cytokines and chemoattractants (141, 142), essential processes for damage repair. Proteases released by activated MCs convert IL-33 to a more active form (143), thereby amplifying the initial effect of IL-33 and attracting more immune cells to migrate to the site of injury. Moreover, activated MCs and DCs produce IL-33 (144, 145), activating specific immune cells to release mediators that stimulate fibroblasts to initiate wound healing (146) and close the gap in the barrier (147–149). Furthermore, IL-33 production is promoted by NOD1 signaling in chronic *H. pylori*-infected gastritis, which prevents excessive inflammation (150). Therefore, IL-33 has a dichotomous role in *H. pylori* infection-related gastric mucosal disease, acting as both an accelerator of disease progression and a key factor in reversing disease exacerbation.

Furthermore, it is interesting that IL33 induces a Th2-biased response. However, the downregulation of IL33 mRNA or IL33 knockout in *H. pylori*-positive human gastric samples and mice with a chronic *H. pylori* infection may lead to Th1/Th17 immune dysregulation during subsequent pathology (65), and both conditions have been shown to evoke precancerous changes (78, 151, 152). These data suggest that chronic *H. pylori*-mediated inhibition of gastric IL33 may be a key event in GC progression, preventing the induction of Th2 immunity and dysregulating the local immune response to Th1/Th17 cells, thereby exacerbating carcinogenesis. Therefore, IL-33 is critical in *H. pylori*-associated gastritis and GC.

## The expression pattern and functional role of HMGB1 in the stomach

The HMG superfamily of nucleosome-binding proteins can be divided into three subfamilies: HMGA, HMGB, and HMGN (73). HMGB is the most common gastric HMG protein, and HMGB1 has been widely studied in gastric diseases (39, 40). Initially, HMGB1 was shown to be a DAMP. Therefore, as an alarmin, HMGB1 can be actively secreted by various inflammatory cells (74), passively released by necrotic and apoptotic cells (75–77, 153, 154), and selectively released through tumor cell autophagy (75, 79). Extracellular HMGB1 activates DCs to promote their functional maturation and stimulates them to secrete HMGB1, thereby maintaining antigen-specific T-cell proliferation, preventing T-cell activation-dependent apoptosis and promoting Th1-skewed differentiation (80). In addition, HMGB1 usually binds to Toll-like receptors (TLRs) or receptors for advanced glycation end products (RAGEs) (81, 82, 155–157) to activate innate immunity (83, 84), thereby participating in tissue repair.

In the stomach under physiological conditions, HMGB1 is mainly localized in the nucleus and cytoplasm of gastric epithelial cells (39) and is involved in transcriptional regulation as a chromatin-binding factor associated with specific DNA-binding proteins (158, 159). HMGB1 is significantly upregulated in gastric mucosal diseases, and upon upregulation, HMGB1 acts as a potent chemokine, triggering the infiltration of inflammatory immune cells and increasing the progression of gastric mucosal diseases (40, 160). Therefore, HMGB1 is a key inflammatory signal in controlling gastric mucosal diseases (160, 161).

### HMGB1 promotes the development of gastric mucosal diseases

#### HMGB1 enhances GC proliferation and metastasis

Early studies showed that HMGB1 levels are highly correlated with the depth of invasion, lymph node metastasis, tumor size and poor prognosis of GC (85). Therefore, a great deal of research has been conducted to understand the relationship between HMGB1 and GC. Initially, some researchers suggested that inhibiting HMGB1 could upregulate Mcl-1 transcription, thereby increasing autophagy and promoting GC cell apoptosis (28), which paved the way for later studies on the role of HMGB1 in GC. Since, researchers have proposed many mechanisms to explain the involvement of HMGB1 in GC proliferation and metastasis, such as the HMGB1-mediated PI3K/Akt/HIF-1α signaling pathway (162) and activation of the MEK/ERK or NF-κB signaling pathway to induce GC cell proliferation through interactions with RAGE (163, 164). Moreover, HMGB1 can also enhance the expression of cyclins, thereby inducing epithelial–mesenchymal transition and matrix metalloproteinase (MMP) expression and promoting the upregulation of RAGE, which activates the Akt/mTOR/P70S6K and ERK/P90RSK/CREB signaling pathways to regulate GC cell proliferation and migration (165). In addition, another study showed that the HMGB1/TLR4/MyD88 signaling pathway promotes GC progression and that silencing HMGB1/TLR4/MyD88 signaling in GC cells with HMGB1 siRNA significantly inhibits GC cell proliferation, migration and invasion and induces apoptosis *via* the NF-κB pathway (86). Moreover, recent studies have shown that exosomes released by GC cells carry HMGB1, which can induce N2 neutrophil polarization through the HMGB1/TLR4/NF-κB signaling pathway, resulting in GC cell proliferation and migration (87). These data suggest that HMGB1 may represent a new therapeutic target in GC.

#### HMGB1 is involved in the progression of GUs

HMGB1 can be activated and released into the extracellular environment through inflammatory stimulation, oxidative stress and other injuries (88). HMGB1 is released into the extracellular environment and stimulates cytokine production through RAGE or TLR4, which triggers inflammation and recruits leukocytes to the site of tissue damage (166). Neutrophils are the most common cell type recruited, and excess neutrophil infiltration is a negative regulator of GU healing (167). Neutrophil extravasation to the injury site increases ROS levels (168), and high ROS levels can damage the gastric mucosal barrier by oxidizing cellular proteins and lipids (169), thereby increasing permeability and leading to inflammation. Moreover, ROS production can stimulate the release of inflammatory cytokines such as TNF-α and NF-κB from macrophages (169). TNF-α blocks gastric microcirculation around the mucosa of the ulcer, further delaying ulcer healing (170).

Moreover, HMGB1 binds to RAGE or TLR4, which can inhibit the phosphorylation and proteasomal degradation of IκBα, releasing NF-κBp65 for transport to the nucleus and thereby activating the proinflammatory NF-κB pathway [6,7] and triggering the transcription of proinflammatory cytokines such as IL-1β and TNF-α (171) to exacerbate gastric ulceration (172, 173). Moreover, reducing gastric oxidative stress can interfere with NF-κBp65 binding to the promoter region of target proinflammatory cytokines and thereby inhibit the redox-sensitive NF-κB pathway (174). Therefore, inhibiting the HMGB1/RAGE pathway may protect against GU injury.

Furthermore, GU induction experiments showed that HMGB1 expression increases in response to activation of the nucleotide-binding domain and leucine-rich repeat protein 3 (NLRP3) inflammasome and NF-κBp65 (175). Previous studies have demonstrated that HMGB1 activates the NLRP3 inflammasome (176) and is involved in a variety of inflammatory diseases (177–179), which may create a positive feedback effect on GU. The NLRP3 inflammasome, an important proinflammatory mediator that is involved in ulcer pathogenesis, is activated by binding to PRRs, which increases the expression of pro-IL-1β and pro-TNF-α (95), thereby damaging the gastric mucosa. Therefore, high HMGB1 expression is one reason for delayed GU healing. When the HMGB1/NLRP3/NF-κB pathway is inhibited, the expression of IL-1β and TNF-α is downregulated, thereby promoting GU healing. However, some researchers hold the opposite view because extracellular HMGB1 and RAGE induce the migration and proliferation of vascular-associated stem cells (angioblasts), which may promote tissue regeneration (96). Moreover, studies have demonstrated that HMGB1 can reduce tissue damage in inflammatory bowel disease and other complex inflammatory diseases by regulating autophagy and apoptosis (97). Therefore, HMGB1 may have dual roles in GUs, both damaging tissue and promoting tissue repair and resisting damage.

## Gastric alarmin AMPs: Defensins and cathelicidin

AMPs are an original immune mechanism, and this class of peptides and small proteins has microbicidal activity. Initially, AMPs were extensively studied in insects and other invertebrates. However, there is growing evidence that AMPs also play a crucial role in human immunity. There are two types of AMPs in human tissues and cells called defensins and cathelicidins, which are mainly produced by epithelial cells and neutrophils (92). Because of the immune effect of defensin and cathelicidin in gastric diseases, they have received increasing attention. AMPs have antimicrobial activity in the stomach, acting as a mucosal defense mechanism at key locations in the mucus layer (93).

### Expression patterns and functional roles of defensins in the stomach

Defensins, including α-, β-, and θ-defensins, are cationic antimicrobial host defense peptides consisting of a characteristic β-sheet and six disulfide-linked cysteines (89, 94). In humans, the defensin subfamily mainly includes α- and β-defensins (18). Of the six human α-defensins, human neutrophil peptides 1-4 (HNP-1, HNP-2, HNP-3 and HNP-4) are mainly produced by neutrophils (90), whereas the best-studied β-defensins (HBDs, HBD1–HBD4) (91) are produced by various epithelial and mucosal cells (98). Human defensins bind to receptors (99, 180, 181), including CC chemokine receptor 2 (CCR2) (182), CCR6 (100), TLR4 (183) and TLR9, to induce chemokine-mediated immune cell migration to sites of tissue damage and thus participate in tissue damage and repair.

Defensins are localized at the cell membranes in the gastric surface epithelium (32, 184), where they participate in host antibacterial defense (29) and coordinate innate and adaptive immunity to maintain gastric mucosal homeostasis (101, 102). Defensin expression is increased in gastric mucosal diseases, including CG, GU, benign gastric polyps (BGPs) and GC (30, 103, 185), and defensins are involved in the progression of gastric mucosal diseases.

### Defensins promote the development of gastric mucosal diseases

#### Defensins are involved in the development of GC

Recent studies have shown that α-defensin 5 (DEFA5) overexpression inhibits the development of GC (186). Mechanistically, DEFA5 induces cell cycle arrest by binding to BMI1, reducing its binding to the CDKN2a locus and upregulating the expression of the cyclin-dependent kinase inhibitors p16 and p19, therefore significantly increasing the number of cells in the G1 phase and inhibiting tumor growth (186). Therefore, DEFA5 may act as a tumor suppressor in GC. Overall, defensins play key regulatory roles in the occurrence of GC.

#### Defensin release caused by *H. pylori*-associated gastric mucosal diseases

Recent studies have shown that defensins, including HNP1-3 and HBD1-4 (30, 187–189), are highly expressed in *H. pylori*-associated gastritis, and this increased expression may represent a defensive response of the gastric mucosal barrier to limit infection (190). However, as bacteria and immunity begin to compete, *H. pylori* virulence factors activate the NLRC4 inflammasome and the NF-κB pathway, resulting in the downregulation of defensin expression (191). Moreover, *H. pylori*-infected cells block interferon β (IFNβ), IL-6 and IL-22 signaling to suppress defensin production (192), which results in increased colonization of the stomach by antibiotic-resistant bacteria (193). These events promote the persistence of bacteria in the gastric niche, leading to gastritis, ulcers and even cancer (188). However, another study reported unexpected results; specifically, the marked increase in defensin HNP1-3 in *H. pylori*-infected patients (30) formed a positive feedback cycle with neutrophils (194). This finding may relate to IL-8 release by *H. pylori*-infected gastric epithelial cells, which stimulates massive neutrophil infiltration (195) and the release of α-defensins (105, 109, 110), with direct toxic effects on tissue cells. Moreover, α-defensins stimulate epithelial cells to secrete IL-8 (106), which further increases gastric inflammation and exacerbates injury (107, 108). Thus, defensins have dual roles in *H. pylori*-associated gastritis: they are involved in protecting the gastric mucosa and can deleteriously promote the recruitment and accumulation of inflammatory cells that mediate the progression of gastritis.

In addition, there is some evidence that defensins are involved in the progression of GC. *H. pylori* infection induces HBD-2 and HBD-3 mRNA expression in human gastric adenocarcinoma cell lines (104, 113), but HBD-2 protein was not detected in specimens from *H. pylori*-negative patients (196). In conclusion, defensins may be novel targets for the treatment of *H. pylori*-associated gastritis and GC.

## Expression pattern and functional role of cathelicidin in the stomach

Cathelicidins are a conserved family of host defense peptides that inhibit microorganisms (110) and modulate immune responses. Typically, cathelicidins are expressed in epithelial cells, lymphocytes and monocytes (110, 111). Cathelicidins use human formyl peptide receptor-like-1 (FPRL1) to induce neutrophil and monocyte migration (112, 114) or activate plasmacytoid and myeloid DCs *via* TLR7, 8 or 9, all of which have been shown to contribute to autoimmune disease and wound healing (197–200). In the cathelicidin family, LL-37/human cationic AMP 18 (hCAP18) is the only member expressed in humans. LL-37 is expressed in the cytoplasm of normal gastric fundic gland surface epithelial cells, chief cells and parietal cells (nuclear or membrane); however, when the gastric mucosa is damaged, hCAP18 activates the innate immune system in the stomach to participate in disease progression (31).

### Cathelicidins promote the development of gastric mucosal diseases

#### Inhibitory effect of cathelicidins on gastric hyperplastic polyps and GC

As the only cathelicidin in humans, LL-37 is absent or expressed at very low levels in gastric hyperplastic polyps and gastric tumors (31, 201). Moreover, exogenous LL-37 can block GC cells in the G0/G1 phase and inhibit cell proliferation, and the deletion of endogenous LL-37 stimulates DNA synthesis in GC cells, indicating its antiproliferative effect. LL-37 reduces the production of cytokines such as TNF-α and IL-6 by activating p44/42 MAPK and controlling IL-32 (202), which may inhibit tumor growth. In addition, LL-37 may inhibit mitosis through a proteasome-dependent mechanism that activates bone morphogenetic protein (BMP) signaling (201). Therefore, LL-37 may function as a tumor suppressor peptide in gastric carcinogenesis. Moreover, studies have demonstrated that inhibiting LL-33 N-formyl peptide receptors (FPR1, FPR2, and FPR3) leads to the inhibition of tumor angiogenesis (31). Therefore, LL-37 is a protective factor in gastric hyperplastic polyps and GC, bringing new hope for anticancer therapeutics.

#### Cathelicidin deficiency promotes the development of *H. pylori*-associated gastric mucosal diseases

Cathelicidin is an important promoter of gastric mucosal repair and mucosal barrier protection (203). Increased production of LL-37/hCAP18 by gastric epithelial cells contributes to host mucosal defense in *H. pylori*-associated gastritis (204). Moreover, cathelicidin can inhibit the growth of *H. pylori*, destroy the bacterial biofilm, and induce morphological changes in the *H. pylori* membrane, thereby resisting damage (203). In addition, studies have shown that cathelicidin-knockout mice exhibit greater *H. pylori* colonization; increased production of the proinflammatory cytokines IL-6, IL-1β and ICAM1; and lower expression of the anti-inflammatory cytokine IL-10, which causes tissue damage (203). In addition, rat studies have shown that the host defense peptide rCRAMP promotes GU healing by directly stimulating the transforming growth factor alpha (TGF-α)-dependent transactivation of epidermal growth factor receptor (EGFR) and the downstream signaling mediators ERK1/2 in epithelial cells of the stomach (205). Therefore, cathelicidin plays an important regulatory role in *H. pylori*-associated gastritis and GU and may be a new target for the treatment of *H. pylori*-associated gastric mucosal diseases.

## Alarmins function as diagnostic markers or therapeutic factors in other immune diseases

Recent studies have revealed that alarmins may be diagnostic markers and therapeutic targets (206–209). For example, the release of the active 18/21 kDa fragments of IL-33 may promote the expansion of proinflammatory signaling, a potential indicator of inflammation in response to damage (206). In addition, studies have shown that the progression of intestinal inflammation can be significantly inhibited by anti-HMGB1 neutralizing antibodies (210, 211), suggesting that host defense peptides may have a role in the development of antibacterial, anti-inflammatory and immunomodulatory therapeutics (212). Alarmins have also been used in studies of other diseases as an inflammatory factor assay (213), prognostic assessment (214), and drug therapy in patients with asthma (215, 216) or atopic dermatitis (217).

## Conclusions

Previous studies have revealed the role of certain alarmins in gastric mucosal repair and have provided convincing evidence that these alarmins promote the proliferation and migration of neighboring cells, recruit various immune cells for antibacterial responses and tissue damage repair, and protect the mucosal barrier. Based on these functions, alarmins are potential therapeutic targets in inflammation and even cancer. However, continued alarmin stimulation can also lead to tissue damage and even cancer. This review provides a basic, systematic summary of gastric alarmins, which will prompt researchers to focus on the functional diversity of alarmins in gastric mucosal diseases and provide new perspectives for both treatment and prevention.

## Author contributions

XL and TL conceived and designed the review. EW drafted the manuscript. ZM participated in the data investigation and analysis. JZ assisted in the preparation of the charts. BT, TL and XL edited and revised the manuscript. ST and KM revised and checked this paper. All authors contributed to the article and approved the submitted version.

## Funding

This research was supported by the National Natural Science Foundation of China (81860103 and 82070536 to X.L., 82160505 and 81660098 to T.L., and 82073087 to B.T.) and the Guizhou Province International Science and Technology Cooperation (Gastroenterology) Base [Qian Ke He Platform Talents-HZJD (2021) 001 to X.L.].

## Acknowledgments

We are grateful to GRW, HJ and JXA, who provided suggestions for the article and supported the daily experiments. Simultaneously, I would like to thank the Collaborative Innovation Center of Chinese Ministry of Education (2020-39) for providing funding.

## Conflict of interest

The authors declare that the research was conducted in the absence of any commercial or financial relationships that could be construed as a potential conflict of interest.

## Publisher’s note

All claims expressed in this article are solely those of the authors and do not necessarily represent those of their affiliated organizations, or those of the publisher, the editors and the reviewers. Any product that may be evaluated in this article, or claim that may be made by its manufacturer, is not guaranteed or endorsed by the publisher.
